# Collaborative Simulation-Based Learning to Develop Competencies in Physical Therapy Students: Randomized Controlled Trial

**DOI:** 10.2196/85442

**Published:** 2026-09-21

**Authors:** Iván Cuyul-Vásquez, Hilda Aravena-Páez, Felipe Araya-Quintanilla, Sergio Guinez-Molinos, Rodrigo Núñez-Cortés, Héctor Gutiérrez Espinoza

**Affiliations:** 1 Departamento de Procesos Terapéuticos, Facultad de Ciencias de la Salud, Universidad Católica de Temuco, Temuco, Chile; 2 Facultad de Ciencias de la Salud, Universidad Arturo Prat, Sede Victoria, Chile; 3 Facultad de Medicina, Departamento de Clínicas, Kinesiología, Universidad Católica del Norte, Coquimbo, Chile; 4 Escuela de Kinesiología, Facultad de Ciencias de la Rehabilitación y Calidad de Vida, Universidad San Sebastián, Santiago, Chile; 5 Laboratorio de Simulación e Informática Biomédica, Facultad de Medicina, Universidad de Talca, Talca, Chile; 6 Department of Physical Therapy, Faculty of Medicine, University of Chile, Santiago, Chile; 7 Faculty of Education, Universidad Autónoma de Chile, Av. Pedro de Valdivia 425, Santiago, Chile, 56 992999298; 8 Universidad Espíritu Santo, Samborondón, Ecuador

**Keywords:** simulation training, patient simulation, low back pain, physical therapy, health education

## Abstract

**Background:**

Although the implementation of simulation-based learning has progressed substantially in medicine, physical therapy education shows a lower level of development regarding standardized collaborative models.

**Objective:**

This study aims to determine the effectiveness of collaborative versus individual simulation-based learning in developing clinical competence in undergraduate physical therapy students in the care of standardized patients with low back pain flare-ups.

**Methods:**

A parallel-group randomized controlled trial was conducted. Thirty-one physical therapy undergraduate students were randomized (1:1) to a collaborative or individual simulation-based learning group. Students underwent 3 weeks of clinical simulation training. Standardized patient assessments were performed before and after the simulation-based learning intervention. Clinical competence was evaluated as the primary outcome, while effective communication and therapeutic alliance were evaluated as secondary outcomes.

**Results:**

For clinical competence, a significant main effect of time was observed (*η*_p_^2^=0.739; *P*<.001), with equivalent pre-to-post improvements in both the collaborative (*Δ*=+6.17; *P*_Bonf_<.001) and individual simulation-based learning groups (*Δ*=+5.39; *P*_Bonf_<.001); the interaction effect was nonsignificant (*P*=.61). For communication, no significant time (*P*=.91) or group × time interaction (*P*=.83) effects were observed. Therapeutic alliance scores demonstrated a significant main effect of time (*η*_p_^2^=0.294; *P*=.008), indicating an overall decrease, though group-specific pre-to-post declines did not survive Bonferroni correction (both *P*_Bonf_≥.11).

**Conclusions:**

Collaborative simulation-based learning effectively develops physical therapy clinical competencies, and its effects are comparable to those of individual simulation-based learning with a standardized patient. However, there were no differences between the groups for effective communication or therapeutic alliance. Further research is warranted to assess the efficacy of these modalities in diverse clinical settings.

## Introduction

Assessment in health care education is critical to guarantee and certify clinical competence, an operational construct integrating cognition, clinical reasoning, communication, and psychomotor skills [1]. In physical therapy, acquiring these competencies depends heavily on clinical context and multidimensional domains, including patient-centric care and interpersonal skills [2]. Particularly, the management of musculoskeletal conditions such as chronic low back pain (CLBP) represents a cornerstone of professional practice [3]. Although low back pain is highly prevalent [4], its clinical course is often nonlinear, manifesting as acute, recurrent “flare-ups” characterized by sudden symptom worsening, severe emotional distress, catastrophizing, and fear-avoidance behaviors [5,6]. Consequently, developing clinical competencies within undergraduate curricula to effectively manage the complex intersection of tissue irritability, cognitive-behavioral barriers, and communication challenges during a CLBP flare-up is an urgent educational necessity.

Simulation-based learning in health care education has been proven to be an effective learning method by providing a similar condition of clinical practice in a controlled and safe environment [7,8]. Simulation-based experiences allow students to reflect and learn from their mistakes in a safe environment, ultimately enhancing patient safety [9,10]. Consequently, simulation-based education has experienced exponential growth [11] and has been used to assess professional competencies related to patient-centric care, practice psychomotor, and teamwork skills [12]. High-fidelity simulation, relying on expensive, technologically advanced manikin simulators or heavily staffed standardized patient programs, presents substantial financial barriers, making wide-scale implementation unsustainable for institutions with limited economic resources [13]. Despite these operational challenges, there is a scarcity of literature establishing how low-cost, high-fidelity simulation frameworks can be optimized to achieve clinical performance outcomes comparable to those of resource-intensive traditional modalities.

Simulation-based learning integrated within a peer-assisted learning framework represents a novel paradigm to address this pedagogical and economic gap [14]. Preliminary studies have demonstrated that students and educators are satisfied with what can be achieved through teamwork between peers, patients, and health care professionals [14-16]. Collaborative simulation-based learning is a structured learning model for the acquisition and assessment of clinical competencies in which small teams of students collaborate to design and perform clinical simulation scenarios [17,18]. In this approach, students occupy the center of the instructional design process, alternating roles between physical therapists, observers, and standardized patients, culminating in instructor-led debriefings [17]. By decentralizing the instructor’s traditional role and eliminating the ongoing financial burden of external professional actors, this collaborative architecture theoretically maximizes contextual and social complexity while minimizing institutional operational costs [17,18]. Crucially, within the context of complex chronic conditions, the collaborative model may optimize student learning by distributing cognitive load, thereby preserving the emotional regulation and communication skills required to manage a challenging patient.

While the implementation of simulation-based learning has progressed substantially in medicine, physical therapy education shows a lower level of development regarding standardized collaborative models [19]. Furthermore, the efficacy of using collaborative simulation-based learning to address highly complex, psychosocially driven clinical scenarios, such as CLBP flare-ups, remains virtually unexamined. Although collaborative architectures are presumed to build clinical competencies cost-effectively, their comparative efficacy against traditional, resource-demanding individual simulations has not yet been established for outcomes that depend directly on interpersonal dynamics, such as patient perception and the therapeutic bond. Robust experimental data are required to determine if student-led collaborative designs compromise or enhance multidimensional outcomes, particularly those metrics highly sensitive to patient-provider interactions. Therefore, the aim of this study was to determine the effectiveness of collaborative versus individual simulation-based learning in developing clinical competence, effective communication, and therapeutic alliance among undergraduate physical therapy students during the care of standardized patients with CLBP flare-ups.

## Methods

### Design

The report of this study followed the recommendations of the Simulation-Based Research Extensions for the CONSORT (Consolidated Standards of Reporting Trials; Checklist 1) Statement [20] and the Modified Medical Education Research Study Quality Instrument [21].

**Figure 1.**
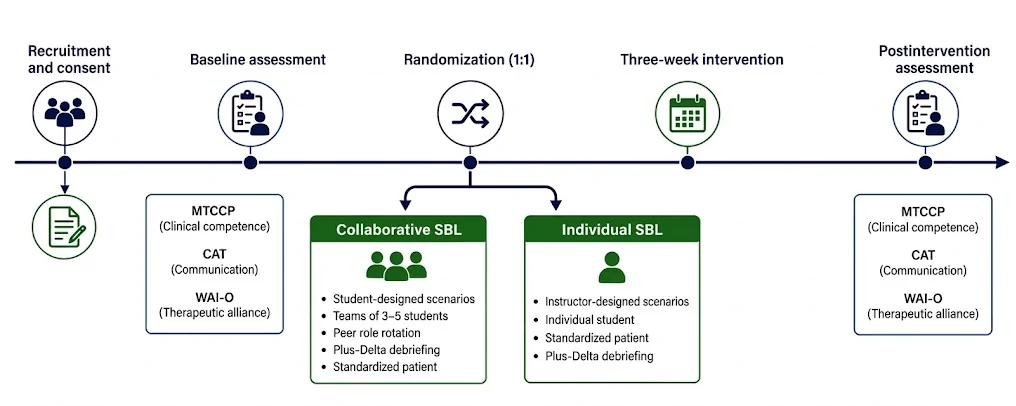
Study design. CAT: Communication Assessment Tool; MTCCP: Measurement Tool for Clinical Competencies in Physiotherapy; SBL: simulation-based learning; WAI-O: Working Alliance Inventory–Observer Version.

This was a parallel-group randomized controlled trial with a pretest-posttest design comparing collaborative and individual simulation-based learning over 3 weeks in undergraduate physical therapy students (Figure 1). Participants were randomly assigned (1:1) to either collaborative or individual simulation-based learning and completed standardized patient assessments before and after the intervention. The learning assessment consisted of an individual standardized patient encounter, from which the primary outcome (clinical competence) and secondary outcomes (communication and therapeutic alliance) were evaluated. The study was conducted in the rehabilitation laboratories of the Faculty of Health Sciences of the Catholic University of Temuco, Chile.

### Recruitment and Eligibility Criteria

Nonprobabilistic convenience sampling was used for this study. All eighth-semester physical therapy students (N=48) enrolled in the Musculoskeletal Rehabilitation course during 2022 were screened for eligibility. The inclusion criterion required the successful completion of the course. An independent researcher, who held no grading responsibilities or administrative ties to the students, conducted an informational session in December 2022. During this meeting, the study objectives and simulation-based experiences were detailed, emphasizing that participation was strictly voluntary and would result in no academic prejudice. Of the 48 eligible students, 31 (64.5%) provided written informed consent and were enrolled. Data collection and clinical simulation training took place between December 2022 and January 2023, coinciding with the start of the summer break.

### Randomization and Blinding

Participants were randomly assigned to either an experimental group with collaborative simulation-based learning or a control group with individual simulation-based learning. A simple computer-based randomization (1:1) was performed via a website [22] by an assistant not associated with the research. The evaluators and the statistical analyst were blinded to group assignment. Allocation concealment was achieved through opaque sealed envelopes. Participants were blinded to the research hypothesis. Due to the nature of this research, the instructor who conducted the simulation-based experience could not be blinded to group assignment.

### Intervention: Simulation-Based Learning

The learning objective was to apply a physical therapy intervention based on exercise and education to a standardized patient with CLBP. Collaborative simulation-based learning (experimental group) was carried out according to the structure proposed by Guinez-Molinos et al [18]: (1) educational design: the creation of the evidence-based guide provided the theoretical framework for students to design, simulate, and evaluate collaborative scenarios; (2) collaborative design: 3 teams of 3 to 5 students were formed. Each team designed a clinical case based on the theme covered in class during the week. Students were also encouraged to write a brief clinical history. Additionally, they assigned specific roles within their simulation scenario. Through this method, weekly active participation was promoted among all students. Each week, the roles rotated among the students in each group; (3) simulation: the student guide explained the clinical simulation procedure to 2 students from another team and controlled the time. Observing students identified opportunities for improvement; (4) debriefing: unlike the protocol proposed by Guinez-Molinos et al [18], a plus-delta debriefing was used [23]. Conversely, the control group underwent individual simulation-based learning, following a traditional structure that used standardized patients. Details of each simulation-based learning protocol are presented in Multimedia Appendix 1.

The procedures for simulation-based learning were standardized into five stages to ensure validity and comparability between the groups:

1. Pre-briefing: The prebriefing procedures were similar for both groups and led by the same instructor. Each week, a 1.5-hour face-to-face lecture on the theoretical content was held, followed by a 30-minute information session dedicated to the simulation-based experience (SBE) procedures and the general history of the standardized patient. In addition, an evidence-based guide for independent study was provided, summarizing the theoretical content related to the rehabilitation of patients with CLBP and acute flare-ups. This guide was included in the lectures [19].
2. Design: This stage lasted 20 minutes for both groups to balance exposure time. In the control group, the scenario design was carried out beforehand by the instructor, who also trained the standardized patients. During this time, the control group students reviewed their notes before the briefing began. In the experimental group, students designed the scenario in teams of 3 to 5 people. This process included creating a brief case history and assigning specific roles: patient, companion, observer, and student guide. The instructor provided the general characteristics of the case to ensure that the diagnosis and duration of pain were comparable to those of the control group. The scenarios aimed to have students confront 3 types of patients with CLBP, presenting with nociceptive, neuropathic, or nociplastic pain. In the final 2 weeks, the scenarios included patients experiencing flare-ups. The characteristics of the scenarios are reported in Table S1 (Multimedia Appendix 2).
3. Briefing: This phase lasted a total of 7 minutes. The instructor presented the fiction contract and psychological safety guidelines, including establishing an atmosphere of trust, normalizing error, maintaining confidentiality, and creating a judgment-free environment. In the control group, the instructor explained the procedure to a randomly selected student, who then had 2 minutes to read the case history posted on the room door (Table S2 in Multimedia Appendix 2). In the experimental group, the student guide from each team was responsible for explaining the procedure to 2 students from another team, followed by a 2-minute reading of the case history, similar to the control group.
4. Scenario: The scenario lasted 10 minutes for both groups. Students had to gather information, reach a consensus, and implement a physical therapy intervention based on exercise and education for a standardized patient with an acute episode of CLBP. In both groups, the performance was observed by the instructor and peers via a private live YouTube stream to avoid interruptions to the scenario. In the control group, the student interacted directly with a standardized patient from a university theater company, who had been previously trained to ensure script consistency. In the experimental group, 2 students from an external team acted as physical therapists and performed the intervention within a scenario created by another group. The team that designed the case provided 2 students with the roles of patient and companion, alongside a student guide and observers.
5. Debriefing: Immediately after the scenario, a debriefing session of approximately 20 minutes was conducted by the same instructor for both groups. The Plus-Delta model was used [23], focusing on the analysis of the learning objective, clinical reasoning, and observed performance. During this phase, the instructor reinforced the continuity of the psychological safety guidelines initially established, encouraging reflection on opportunities for improvement and the successes achieved during the intervention.

### Data Collection and Outcomes

Demographic data, such as age, sex assigned at birth (female or male), work, and number of courses, were collected in an online form. As potential confounder variables, the Academic Stressors Scale, the Academic Self-Efficacy Scale, and the R-14 Academic Resilience Scale were included in the online form.

All students were assessed individually before and after a 3-week simulation-based learning intervention (either collaborative or individual). The assessment consisted of a simulation-based scenario involving standardized patients. Clinical competency was defined as the primary outcome, while communication and therapeutic alliance served as secondary outcomes. Like other studies [24,25], students’ patient-centered communication skills were evaluated by standardized patients during the scenario; these patients had been previously trained to use the specific evaluation instrument. To minimize recall bias, the standardized patients completed their evaluations immediately following each encounter.

Consistent with previously published protocols, clinical competence and therapeutic alliance were evaluated via video recordings by independent physical therapists who were unfamiliar with the students and remained blinded to group assignments [26]. All students’ clinical performances were recorded using high-definition video and audio. To facilitate this, a camera equipped with an ambient microphone was positioned 3 m from the simulation scenario. This setup allowed for subsequent independent assessment by physical therapists with 5 years of experience in musculoskeletal rehabilitation. Prior to the assessment, these evaluators completed a 3-hour training session on the application of the instruments used to measure therapeutic alliance and clinical competence. This training involved evaluating 8 pilot video recordings, reviewing discrepancies, and receiving feedback from the principal investigator.

### Learning Assessment

The pre- and post-intervention evaluations at the simulation-based scenario lasted 12 minutes, during which each student performed an intervention for a standardized patient experiencing a CLBP flare-up. Two clinical cases were developed to evaluate postintervention learning outcomes in both groups. A clinical case was used in the initial assessment, and another in the final assessment (Table S3 in Multimedia Appendix 2). The initial clinical characteristics were maintained to make the cases comparable, and only factors such as name, occupation, and family or occupational context were modified. To prepare the standardized patient and the physical space of the scenarios, the same training and review procedures were used as in the simulation-based learning group. In addition, in the training of the standardized patient, it was explained how to evaluate the communication of the students through a validated questionnaire.

### Primary Outcome: Clinical Competence

This was evaluated with the Measurement Tool for Clinical Competencies in Physiotherapy (MTCCP), a valid and reliable (*α*=.98; intraclass correlation coefficient [ICC]=0.9) instrument to assess clinical competency in real and educational physiotherapy settings [27]. The MTCCP consists of 20 items, grouped into 2 subscales: professional behavior and clinical reasoning. The MTCCP uses a discrete measurement scale from 1 to 5. Those MTCCP items applicable to the simulated scenarios were selected for this research. Eight elements were considered to evaluate the video recordings (4 on the professional behavior subscale and 4 on the clinical reasoning subscale). Therefore, the maximum MTCCP score for this investigation was 40 points. A higher score indicates a better command of clinical competence, while lower scores indicate a worse command of clinical competence.

### Secondary Outcomes

#### Communication

This was evaluated using the Communication Assessment Tool (CAT). The CAT is a valid and reliable instrument (*α*=.93) to assess real or standardized patients’ perception of student patient-centered communication skills [24]. The CAT consists of 14 items grouped in 1 dimension and uses a discrete measurement scale from 1 to 5. The standardized patients answered the CAT with regard to each student at simulation-based assessment. A higher score indicates excellent communication, whereas lower scores indicate poor communication.

#### Therapeutic Alliance

This was evaluated with the observer version of the Working Alliance Inventory–Observer Version (WAI-O) [26]. WAI-O is a valid and reliable (internal consistency=0.98; ICC=0.92) instrument used to measure the therapeutic alliance within a session [28,29]. The WAI-O consists of 36 items grouped into 3 dimensions: bond, goals, and tasks. It uses a discrete measurement scale from 1 to 7. A higher score indicates a stronger therapeutic alliance, whereas lower scores indicate a weaker therapeutic alliance.

### Statistical Methods

The sample size calculation was based on a power analysis “a priori compute required sample size*”* with an effect size (*d*=1.85) extracted from a previous and relevant study [30]. With an alpha of .05, power (1- β) of 95%, and Student *t* test, a minimum of 9 participants per group are needed. This sample estimate was increased by 20% after allowing for potential withdrawals, giving 11 participants in each group. The sample size calculation was performed using the G*Power 3.1.9.2 software (Department of Psychology at Heinrich Heine University Düsseldorf).

Descriptive statistics were used to describe the demographic and clinical characteristics of the participants. Continuous variables were the mean and SD, and categorical variables were the number and percentage.

Prior to inferential analyses, the tenability of the assumptions underlying the ANOVA model was systematically evaluated. The normality of each dependent variable was assessed within each group and time point using the Shapiro-Wilk test, supplemented by visual inspection of histograms and normal probability plots (Q-Q plots). The homogeneity of variances was assessed at each time point for all 3 outcomes using the Levene test. All contrasts were nonsignificant (*P*>.05). Given that all participants were assessed at both pre-intervention and post-intervention time points, data were analyzed using a 2 × 2 mixed-design ANOVA (also termed split-plot ANOVA), with group as the between-participants factor and time (pre vs post) as the within-participants (repeated measures) factor. Effect sizes were quantified using 2 complementary indices. Partial eta squared (*η*_p_²) was computed for each source of variance in the mixed ANOVA model and interpreted according to the benchmarks: small (*η*_p_²≈0.01), medium (*η*_p_²≈0.06), and large (*η*_p_² ≥0.14) [31]. Cohen *d* was additionally calculated for all planned post hoc comparisons as the standardized mean difference (ΔM/SDΔ for within-group paired comparisons; ΔM/SDpooled for between-group independent comparisons) and interpreted as small (*d*≈0.20), medium (*d*≈0.50), and large (*d*≥0.80) [32]. Given the exploratory nature of the pairwise comparisons and the priori enumerable set of 4 contrasts per outcome, post hoc analyses were conducted using paired-samples Student *t* tests for within-group (pre-to-post) comparisons and independent-samples Student *t* tests for between-group comparisons at each time point. To control the familywise type I error rate, all *P* values were adjusted using the Bonferroni correction (α’=.05/4=.0125 per comparison). The Bonferroni procedure was selected on the grounds of its conceptual transparency, conservative control of familywise error, and suitability for a small, prespecified set of contrasts [33]. Statistical analyses were performed using IBM SPSS Statistics for Windows (version 26; IBM Corp.).

### Ethical Considerations

This study was approved by the Research Ethics Committee of the Catholic University of Temuco (ID: 091302/22) and prospectively registered at OSF.io [34]. The study was conducted in accordance with the ethical principles of the Declaration of Helsinki. All participants were informed of the study procedures, the voluntary nature of participation, and their right to withdraw at any time without penalty. Written informed consent was obtained from all participants prior to enrollment. Privacy and confidentiality were maintained throughout the study. The participants in this study did not receive any form of compensation for their participation.

## Results

### Baseline Characteristics of Physical Therapy Students

**Figure 2.**
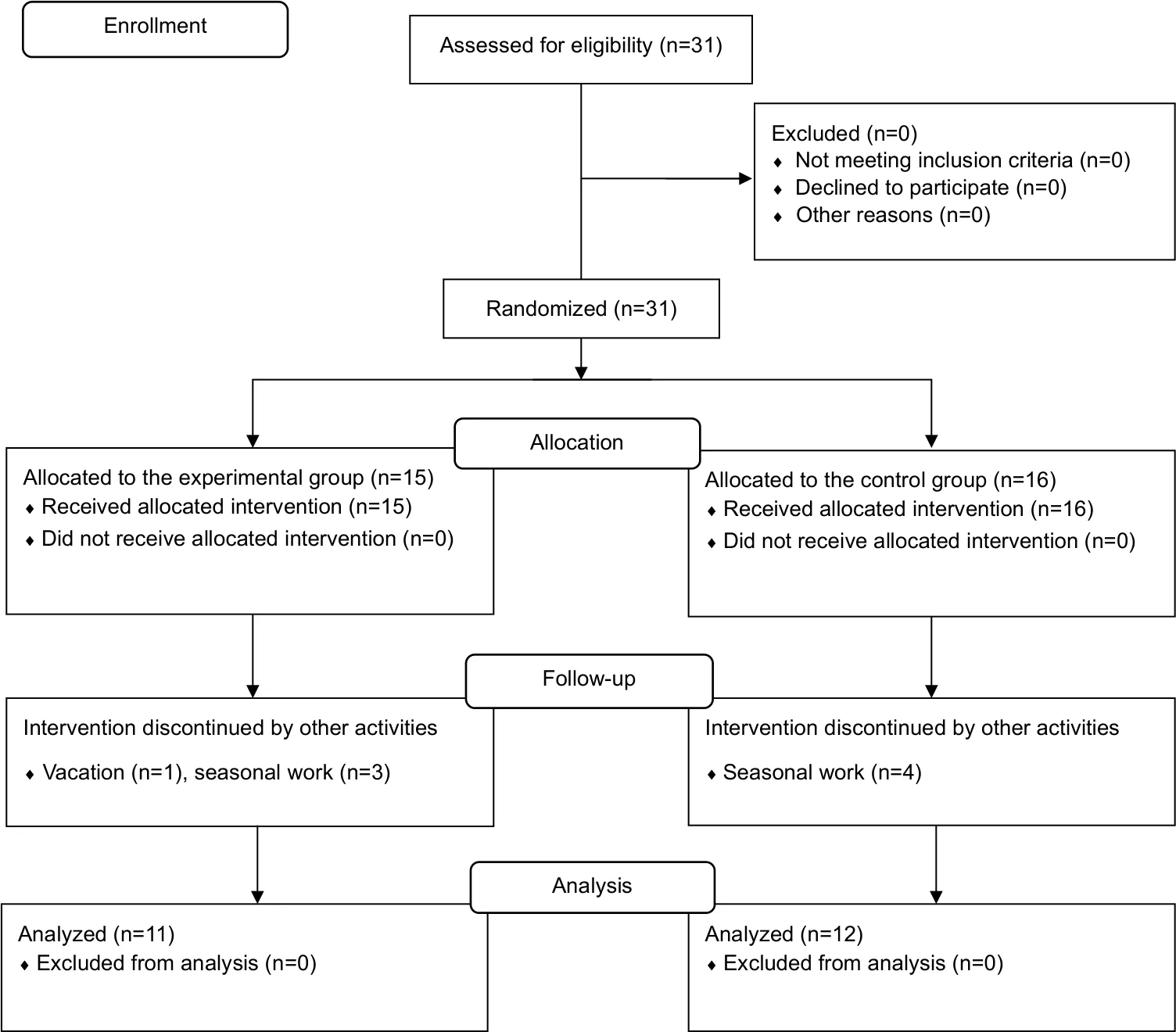
CONSORT (Consolidated Standards of Reporting Trials) 2010 flowchart.

The participants’ timeline can be seen in the flow diagram (Figure 2). A total of 31 students agreed to participate in this research. After randomization, 16 students were assigned to the individual group and 15 to the collaborative group. Eight students dropped out of the study: 1 due to vacation and 7 due to seasonal work. Therefore, 11 students in the individual group and 12 in the collaborative group completed the study. The characteristics of the participants are shown in Table 1. None of the students had previous experience in simulation-based learning with standardized patients. No differences were observed in physical therapy student characteristics at the beginning of the study. Furthermore, no differences were found between groups at baseline for clinical competence (*P*=.42), effective communication (*P*=.16), and therapeutic alliance (*P*=.44).

**Table 1.** Physical therapy students’ characteristics^a^.

Variable	Collaborative (n=12)	Individual (n=11)	Test statistic (*df*)	*P* value
Demographics				
Age (y), mean (SD)	22.6 (1.4)	23.4 (2.7)	−0.886^b^ (21)	.39
Female sex, n (%)	9 (75.0)	8 (72.7)	0.02^c^ (1)	.90
Outcome variables at baseline, mean (SD)				
MTCCP^d^ total score	25.14 (4.33)	26.61 (4.15)	−0.827^b^ (21)	.42
CAT^e^ mean score	4.30 (0.39)	4.53 (0.36)	−1.470^b^ (21)	.16
WAI-O^f^ total score	117.69 (6.26)	120.09 (8.30)	−0.786^b^ (21)	.44

a Between-group comparisons were performed using independent-samples Student *t* tests for continuous variables and Pearson’s *χ*2 test for categorical variables.

b *t* test.

c Chi-square test.

d MTCCP: Measurement Tool for Clinical Competencies in Physiotherapy.

e CAT: Communication Assessment Tool.

f WAI-O: Working Alliance Inventory–Observer Version.

**Figure 3.**
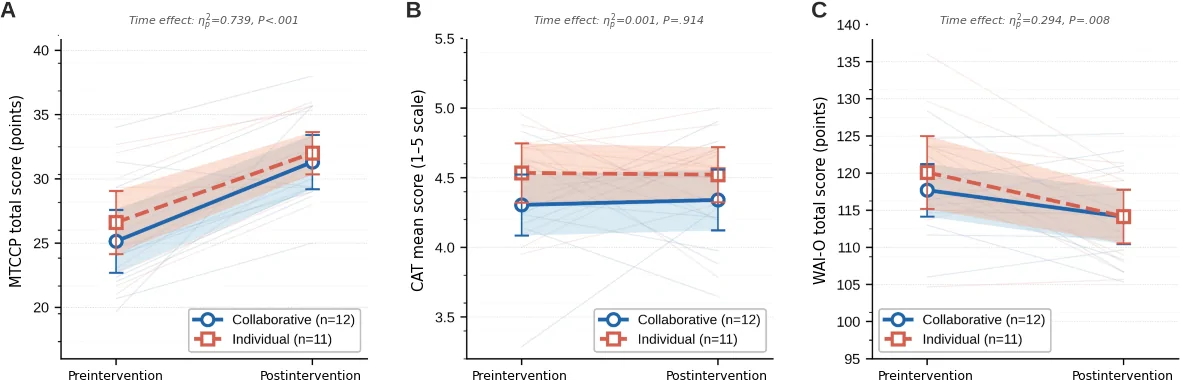
Group × time interaction plots for Measurement Tool for Clinical Competencies in Physiotherapy (MTCCP), Communication Assessment Tool (CAT), and Working Alliance Inventory–Observer Version (WAI-O). Estimated marginal means + 95% CI; individual trajectories shown in translucent lines. (A) Results for clinical competence (MTCCP), (B) results for communication (CAT), and (C) results for therapeutic alliance (WAI-O).

The inferential results from the mixed ANOVA, the detailed descriptive statistics for all outcomes, and their corresponding interaction trajectories are summarized in Tables 2 and 3 and illustrated in Figure 3. Additionally, the complete results for the Bonferroni-corrected post hoc pairwise comparisons are provided in Table S4 (Multimedia Appendix 2).

**Table 2.** Results of the 2 × 2 mixed analysis of variance for Measurement Tool for Clinical Competencies in Physiotherapy (MTCCP), Communication Assessment Tool (CAT), and Working Alliance Inventory—Observer Version (WAI-O)^a^.

Outcome and source of variance	*F* test (*df*)	*P* value	*η*_p_²
MTCCP			
Group	0.595 (1, 21)	.45	0.028
Time	59.47 (1, 21)	*<*.001	0.739
Group × time	0.264 (1, 21)	.61	0.012
CAT			
Group	3.906 (1, 21)	.06	0.157
Time	0.012 (1, 21)	.91	0.001
Group × time	0.046 (1, 21)	.83	0.002
WAI-O			
Group	0.266 (1, 21)	.61	0.013
Time	8.744 (1, 21)	.008	0.294
Group × time	0.546 (1, 21)	.47	0.025

a A mixed-design ANOVA was conducted with group (collaborative vs individual) as the between-participants factor and time (pre vs post) as the within-participants factor. The group × time interaction constitutes the primary inferential test of differential efficacy. *η*_p_² = partial eta squared (Cohen, 1988 benchmarks: small≈0.01, medium≈0.06, large ≥0.14).

**Table 3.** Descriptive statistics for primary outcome variables by group and time point.

Outcome and group	Preintervention, mean (SD)	Postintervention, mean (SD)	Mean change (95% CI)	% Change
MTCCP^b^				
Collaborative	25.14 (4.33)	31.31 (3.73)	+6.17 (3.77 to 8.57)	+24.5
Individual	26.61 (4.15)	32.00 (2.78)	+5.39 (3.38 to 7.40)	+20.3
CAT^c^				
Collaborative	4.30 (0.39)	4.34 (0.39)	+0.04 (−0.35 to 0.43)	+0.8
Individual	4.53 (0.36)	4.52 (0.33)	−0.01 (−0.27 to 0.25)	−0.3
WAI-O^d^				
Collaborative	117.69 (6.26)	114.11 (6.49)	−3.58 (−9.40 to 2.24)	−3.0
Individual	120.09 (8.30)	114.15 (6.10)	−5.94 (−11.12 to −0.76)	−4.9

a Mean change = Post − Pre. 95% CI = 95% confidence interval of the mean change (paired). % Change = (Post − Pre)/Pre × 100.

b MTCCP: Measurement Tool for Clinical Competencies in Physiotherapy.

c CAT: Communication Assessment Tool.

d WAI-O: Working Alliance Inventory–Observer Version.

### Clinical Competence

The mixed ANOVA revealed a large main effect of time on MTCCP scores (*F*_1,21_=59.47, *η*_p_²=0.739; *P*<.001), demonstrating substantial pre-to-post improvement in clinical competencies across both groups. The main effect of group was nonsignificant (*F*_1,21_=0.595, *η*_p_²=0.028; *P*=.45), and the group × time interaction did not reach statistical significance (*F*_1,21_=0.264, *η*_p_²=0.012; *P*=.61). However, the direction and magnitude of improvement were indicative of descriptive parity between conditions. Bonferroni-corrected post hoc analyses confirmed significant pre-to-post gains in both the collaborative group (*t*_11_=−5.231, *d*=-1.510; *P*<.001 [corrected]) and the individual group (*t*_10_=−5.979, *d*=1.803; *P*<.001 [corrected]). Between-group comparisons at preintervention (*d*=−0.346) and postintervention (*d*=−0.211) were small to moderate but nonsignificant after Bonferroni correction (both *P*_Bonf_=1.00), reflecting a high degree of uncertainty due to the limited sample size.

### Communication

For CAT scores, the mixed ANOVA yielded a negligible main effect of time (*F*_1,21_=0.012, *η*_p_²=0.001; *P*=.91), and the group × time interaction was nonsignificant (*F*_1,21_=0.046, *η*_p_²=0.002; *P*=.83). However, the main effect of group was characterized by a large effect size (*F*_1,21_=3.906, *η*_p_²=0.157; *P*=.06), despite missing the conventional threshold for statistical significance. Descriptively, the individual group scored marginally higher. Given that the large effect size (*η*_p_²=0.157) alongside the nonsignificant *P* value, this trend strongly suggests a meaningful descriptive difference between the groups that cannot be conclusively confirmed or ruled out due to statistical underpowering.

### Therapeutic Alliance

The mixed ANOVA indicated a moderate-to-large main effect of time for WAI-O scores (*F*_1,21_=8.744, *η*_p_²=0.294; *P*=.008), reflecting a decrease in observer-rated working alliance from pre- to post-intervention across both conditions. The main effect of group (*F*_1,21_=0.266, *η*_p_²=0.013; *P*=.61) and the group × time interaction (*F*_1,21_=0.546, *η*_p_²=0.025; *P*=.47) were not statistically significant. Post hoc analyses revealed that the pre-to-post decline did not survive Bonferroni correction in either the collaborative group (*t*_11_=1.637, *d*=−0.472; *P*_Bonf_=.52) or the individual group (*t*_10_=2.558, *d*=−0.771; *P*_Bonf_=.11). Nevertheless, the moderate and large individual effect sizes reveal a descriptive reduction in alliance scores across both conditions, suggesting that true underlying changes may be obscured by statistical underpowering.

## Discussion

### Principal Findings

This study aimed to determine the effectiveness of collaborative versus individual simulation-based learning in developing clinical competence, effective communication, and therapeutic alliance among undergraduate physical therapy students during the care of standardized patients with CLBP flare-ups. Within the constraints of our sample size, our findings suggest that both simulation-based learning modalities led to within-group increases in clinical reasoning and professional behaviors, although no statistically significant differences were detected between the 2 modalities in clinical competence. However, observer-rated therapeutic alliance demonstrated a significant main effect of time, representing an overall decline across the intervention in both groups, while effective communication scores did not show statistical differences either between the groups or over time.

To the best of our knowledge, this study represents the first randomized experimental trial directly contrasting student-led collaborative scenario design against investigator-led individual simulation within physical therapy entry-to-practice curricula. The primary novelty of our work resides not merely in the logistical or financial advantages of collaborative simulation-based learning, but in providing preliminary empirical insight into a peer-assisted learning model for managing complex, psychosocially driven clinical conditions. Nevertheless, given the limited statistical power and the high uncertainty surrounding the resulting effect sizes, these findings should be considered exploratory rather than a definitive validation of the model’s magnitude of effect.

Several studies have corroborated the effectiveness of simulation-based learning for the development of physical therapy competencies involving practitioner skills, empathy, motivation, and attitudes [35-37]. While collaborative simulation-based learning has been applied in cardiology emergency scenarios [16], where the perceived usefulness among students and teachers was very high, its comparative effectiveness has not yet been established. Our results suggest that collaborative simulation-based learning may serve as an effective and comparable alternative to individual simulation-based learning with standardized patients. Collaborative simulation-based learning introduces a paradigm shift rooted in social constructivism and cognitive load theory [17,38,39]. By placing students at the center of the instructional design process, requiring them to collaboratively analyze the literature, cocreate clinical scripts, and enact the patient role, the cognitive load associated with complex clinical decision-making is functionally distributed [17,18]. To successfully construct an authentic CLBP flare-up scenario, student dyads must actively negotiate and internalize what a patient experiencing centralized pain, catastrophizing, and fear-avoidance actually undergoes [5,6]. This metacognitive phase prior to execution could explain why the collaborative group achieved clinical competence scores similar to those of the individual group.

Regarding effective communication and the therapeutic alliance, both variables showed no significant differences between groups at the end of the study. While communication remained relatively stable over time, surprisingly, the therapeutic alliance decreased significantly between the pre- and post-intervention periods. Rather than reflecting a failure in teaching, this systematic reduction in the quality of the therapist-patient bond reflects a well-documented phenomenon in medical education, the cognitive rigidity of the novice physical therapist when faced with clinical complexity [40-42]. A flare-up of CLBP generates intense emotional distress and frustration in patients, which in turn complicates the relational context during the clinical encounter [5,6]. High-fidelity and complex standardized patient simulations can potentially induce significant anxiety and negative emotions in undergraduate students. These emotional responses may consume working-memory resources by increasing extraneous cognitive load, potentially hindering learning if total cognitive load exceeds working memory capacity [43,44]. Consequently, to preserve performance, students frequently retreat into a defensive, more technical, and highly prescriptive biomedical mode, focusing strictly on the physical execution of manual techniques or exercise parameters while neglecting affective validation, active listening, and the encouragement of positive expectations [45,46]. Given that an enhanced therapeutic alliance and effective communication are contextual factors capable of significantly reducing pain intensity [47], this procedural limitation represents a critical clinical error.

As not all students can participate in the clinical experience of meeting and interacting with this spectrum of patients, simulating a high-fidelity scenario provides an opportunity to practice and achieve clinical competencies in a safe learning environment. An example of a complex scenario is the management of a flare-up in patients with CLBP, as it impacts major elements of quality of life and involves multidimensional factors, such as a sense of disablement and shifts in mood or emotions, that the student must manage [5,6,48]. Active therapy with a patient-centered approach can modulate the patient’s outcome through communication strategies such as active listening, empathy, building positive expectations, and providing reassuring information [45]. Therefore, promoting a therapeutic alliance and effective communication, while taking into account contextual factors, can be implemented as a key clinical education strategy within the university curriculum of health care professionals [45]. Through either collaborative or individual simulation-based learning, real-life experiences are represented in a guided and interactive manner; however, a collaborative approach may reduce overall costs of simulation, specifically with script development and the cost of actors [49].

### Recommendations for Education

To integrate collaborative simulation-based learning into physical therapy or other health sciences curricula, several guidelines are recommended. First, it is necessary to identify the course, learning outcomes, and competencies to be developed to determine how many collaborative SBEs will be integrated. Second, the instructor must provide reading material that serves as the baseline input for students to create their clinical cases and scenarios. Third, students should be informed in advance of the simulation modality and their specific responsibilities. Fourth, during the collaborative simulation-based learning design phase, the instructor must facilitate the creative process by resolving doubts, defining roles, and reviewing the clinical coherence of the scenario and case against the intended learning outcome (eg, the general characteristics of the patient, presence of a companion, signs, symptoms, psychosocial factors, equipment, and spatial layout). Fifth, the execution of the scenarios is the sole responsibility of the designated student team, requiring members to fulfill the roles of patient, companion, timekeeper, and performance evaluator. Sixth, the debriefing phase can be adapted according to the methodology that best suits the instructor guiding the process.

From an educational curriculum standpoint, these preliminary findings suggest that collaborative simulation-based learning should not be restricted to an introductory method or a contingency plan for low-resource settings. Instead, it should be strategically integrated as a scaffolded instructional methodology. We recommend implementing collaborative peer-designed simulation during early entry-to-practice phases to build solid clinical reasoning and communicative baselines in psychologically safe, peer-supported environments. Once these distributed cognitive frameworks are consolidated, students can progressively transition toward individual encounters with professional standardized patients, culminating in immersion with real, complex clinical populations.

### Strengths and Limitations

To the best of our knowledge, this is the first comparative experimental study directly evaluating the effectiveness of collaborative versus individual simulation-based learning. Following a previously defined and easily reproducible protocol, we trained standardized patients for both the evaluation phases and the simulation-based learning interventions. The assessment sessions were video recorded to prevent students from experiencing heightened stress due to the physical presence of an in-room evaluator. Finally, the evaluation incorporated the dual perspectives of the standardized patient and a blinded external evaluator. However, our findings should be interpreted with caution due to several limitations. Although effective communication and the therapeutic alliance were evaluated, these relational skills were not explicitly included as formal content or core competencies to be developed during SBEs. Furthermore, while we performed a priori sample size calculation based on the existing literature, our final sample size limited the statistical power of the study. In underpowered educational research, these constraints can lead to overinflated effect sizes, a phenomenon that introduces a large amount of uncertainty regarding the true magnitude of the intervention’s impact. Consequently, the unusually high effect sizes observed in our results must be interpreted with caution, as they may restrict the generalizability of the findings to broader physical therapy curricula. Future studies should incorporate larger and more diverse sample sizes, incorporate explicit post hoc power analyses, assessments of student stress and anxiety, and cost-effectiveness analyses, alongside qualitative evaluations of the collaborative simulation experience. Because this study included only physical therapy students, further research is warranted across other health professions, including interprofessional approaches.

### Conclusions

In a complex clinical management context, both collaborative and individual simulation-based learning led to increases in physical therapy clinical competencies, with no statistically significant differences detected between the 2 instructional modalities. However, there were no differences between the groups for effective communication or therapeutic alliance. Given the high magnitude of improvement observed across both modalities, detecting a statistically significant differential effect between them would require larger sample sizes. Further research is warranted to assess the efficacy of these modalities in diverse clinical settings.

## Supplementary material

10.2196/85442 Multimedia Appendix 1 Individual and collaborative simulation-based learning protocols.

10.2196/85442 Multimedia Appendix 2 Additional tables included in the supplementary material.

10.2196/85442 Checklist 1 CONSORT checklist.

## Abbreviations

CAT: Communication Assessment Tool
CLBP: chronic low back pain
CONSORT: Consolidated Standards of Reporting Trials
ICC: intraclass correlation coefficient
MTCCP: Measurement Tool for Clinical Competencies in Physiotherapy
SBE: simulation-based experience
WAI-O: Working Alliance Inventory–Observer Version
