# Correction: Collaborative Simulation-Based Learning to Develop Competencies in Physical Therapy Students: Randomized Controlled Trial

**DOI:** 10.2196/112897

**Published:** 2026-10-30

**Authors:** Iván Cuyul-Vásquez, Hilda Aravena-Páez, Felipe Araya-Quintanilla, Sergio Guinez-Molinos, Rodrigo Núñez-Cortés, Héctor Gutiérrez Espinoza

**Affiliations:** 1 Departamento de Procesos Terapéuticos, Facultad de Ciencias de la Salud, Universidad Católica de Temuco, Temuco, Chile; 2 Facultad de Ciencias de la Salud, Universidad Arturo Prat, Sede Victoria, Chile; 3 Facultad de Medicina, Departamento de Clínicas, Kinesiología, Universidad Católica del Norte, Coquimbo, Chile; 4 Escuela de Kinesiología, Facultad de Ciencias de la Rehabilitación y Calidad de Vida, Universidad San Sebastián, Santiago, Chile; 5 Laboratorio de Simulación e Informática Biomédica, Facultad de Medicina, Universidad de Talca, Talca, Chile; 6 Department of Physical Therapy, Faculty of Medicine, University of Chile, Santiago, Chile; 7 Faculty of Education, Universidad Autónoma de Chile, Av. Pedro de Valdivia 425, Santiago, Chile, 56 992999298; 8 Universidad Espíritu Santo, Samborondón, Ecuador

In “Collaborative Simulation-Based Learning to Develop Competencies in Physical Therapy Students: Randomized Controlled Trial” [1], a conflicts of interest declaration was omitted.

The Conflicts of Interest statement has been added as follows:

*SG-M serves on the JMIR Publications Editorial Board as an associate editor of JMIR Medical Education. However, he was not involved in the review process for this manuscript.*

The correction will appear in the online version of the paper on the JMIR Publications website together with the publication of this correction notice. Because this was made after submission to PubMed, PubMed Central, and other full-text repositories, the corrected article has also been resubmitted to those repositories.
