# Supplementary material for: Collaborative Simulation-Based Learning to Develop Competencies in Physical Therapy Students: Randomized Controlled Trial
Source: JMIR Med Educ. 2026 Sep 21;12:e85442. doi: 10.2196/85442 (PMC13592459; doi:10.2196/85442)
Supplement: Supplementary file 2 [file mededu-v12-e85442-s001.docx]

**Table 1**. Individual and collaborative simulation-based learning protocols.

| Phase | Individual simulation-based | Collaborative simulation-based |
| --- | --- | --- |
| Pre-briefing | The pre-briefing procedures were similar for both groups and were led by the same instructor. Each week, a 1.5-hour face-to-face lecture was held on the content to be applied in the SBE. At the end of each lecture, a 30-minute information session was held on the SBE procedures and the SP general history. The content from the three weeks was summarized in an evidence-based guide for independent study. | |
| Design*  (20 minutes) | To balance the time with the other group, students reviewed their notes for 20 minutes before the briefing. Previously, the instructor designed the scenario and trained a standardized patient. | Students designed the scenario in teams of 3–5 people over a 20-minute period. This process involved creating a brief medical history on a sheet of paper and assigning roles (patient, companion, observer, student-guide). |
| Briefing*  (7 minutes) | The instructor explained the procedures, presented the fiction contract and psychological safety guidelines (atmosphere of trust, normalization of error, confidentiality, judgment-free environment, and mitigation of disruptive behaviors), and randomly selected the student who would play the role of PT (5 minutes). The instructor explained the simulation procedure to the selected student. Reading of a brief clinical history on the door (2 minutes). | The instructor explained the procedures, presented the fiction contract and psychological safety guidelines (atmosphere of trust, normalization of error, confidentiality, judgment-free environment, and mitigation of disruptive behaviors), and randomly selected two students who would play the role of PT (5 minutes). The student-guide explained the simulation procedure to two students from another team. Reading of a brief clinical history on the door (2 minutes). |
| Scenario*  (10 minutes) | SBE sessions were conducted in a rehabilitation room equipped with a stretcher, footstool, and exercise equipment, including elastic bands, kettlebells, dumbbells, cones, coordination ladders, and mats. Prior to each scenario, the equipment and furniture were arranged in a standardized layout for all groups. | |
|  | The student in the role of PT performed the SP intervention (10 minutes). Students had to gather information, reach a consensus, and implement an intervention for a patient experiencing an acute flare-up of CLBP. Peers and the instructor observed via a private YouTube broadcast | Two students from an external team in the role of PT performed the SP intervention (10 minutes). Students had to gather information, reach a consensus, and implement an intervention for a patient experiencing an acute flare-up of CLBP based on the clinical case designed and simulated by the peer team, under the time control of the student-guide. Peers and the teacher observed via a private YouTube broadcast. |
| Debriefing*  (20 minutes)* | The debriefing procedures were similar for both groups and were led by the same instructor. The Plus-Delta model was directed by the instructor. Debriefing focused on clinical reasoning and performance. The instructor reinforced the continuity of the psychological safety guidelines during the debriefing. | |

**Abbreviations**. CLBP: Chronic Low Back Pain, SBE: Simulation-Based Experience, SP: Standardized Patient, PT: Physical Therapist. * These phases were carried out during the SBE and in the same location for both groups.
