# Supplementary material for: Collaborative Simulation-Based Learning to Develop Competencies in Physical Therapy Students: Randomized Controlled Trial
Source: JMIR Med Educ. 2026 Sep 21;12:e85442. doi: 10.2196/85442 (PMC13592459; doi:10.2196/85442)
Supplement: Supplementary file 3 [file mededu-v12-e85442-s002.docx]

**Supplementary material**

**Table S1**. Theoretical contents of Module II of the course

| Weeks | Contents Characteristics |
| --- | --- |
| First | Lumbar flexion movement impairment syndrome of the low back associated to lumbar disc herniation |
| Second | Lumbar extension movement associated to acute lumbar facet joint pain |
| Third | Lumbar complex patient with diverse areas involve. Fibromyalgia was used as an example |

**Table S2**. Characteristics of the clinical scenarios and related domains of physiotherapist practice competence

| Scenario | Clinical history and instructions posted on the door | Domains of physiotherapist practice competence |
| --- | --- | --- |
| Flare-ups  Low back pain | You are going through a clinical experience at a private clinic in which your instructor asks you to **prescribe and dose a exercise** for a client with a diagnosis of Fibromyalgia who is currently being treated for low back pain flare-ups. This is the first physical therapy session, in which the only intervention has been interferential current in conjunction to hot-moist compresses for 20 minutes. | **Domain 1. Physiotherapy assessment and plan intervention:** safely and effectively implement physiotherapy interventions, to restore integrity of body systems essential to movement; to maximize function and recuperation; to minimize incapacity; and to enhance quality of life, physical and mental health, wellbeing, independent  living and workability in individuals and groups with altered movement behaviors resulting from impairments, activity limitations, participatory restrictions or disabilities through therapeutic exercise  **Domain 2. Ethical and professional practice:** practice using a culturally-competent, person-centered approach with respect for all forms of inclusion, diversity, dignity, privacy, autonomy, and human rights of the client, who is seeking services provided in person.  **Domain 3. Communication:** communicate clearly, accurately, understandably, effectively in a culturally-competent manner to create trust and an appropriate environment for physiotherapy intervention, empowerment, and collaboration to enable good outcomes in person . |

**Table S3**. Clinical Case

**Clinical Case for the Student**

You are a physical therapy student on a clinical placement at a rehabilitation clinic. Today, your supervisor has tasked you with designing and prescribing an exercise program for a patient diagnosed with fibromyalgia who is also being treated for non-specific low back pain. This is the patient's first treatment session with you. Just before this session, the patient received a 20-minute application of interferential current and moist hot packs.

**Clinical Case for the Standardized Patient**

**Demographics:**

- **Gender:** Male/Female
- **Age:** 35-45 years old
- **Occupation:** Owner/operator of a local neighborhood store
- **Nationality:** Chilean (from Coquimbo/Temuco)
- **Marital Status:** Divorced
- **Living Situation:** Lives alone with their two children: Matías (10) and Gabriela (17).
- **Socioeconomic Status:** Financially strained; their income is insufficient and depends entirely on the hours they can work.

**Name:** Daniel (if male actor); Daniela (if female actor).

**Reason for Consultation:** Acute flare-up of non-specific low back pain.

**Clinical History:** The patient has a 5-year history of fibromyalgia and chronic pain. In addition to the symptoms from their fibromyalgia, they developed severe low back pain 7 months ago with no apparent mechanism of injury. Functionally, they have difficulty bending over, picking objects up from the floor, and stocking the high shelves at their store. They are concerned their job is worsening the pain but cannot afford to stop working, as the business is the family's only source of income. Their children are in school and unable to provide support.

Two days ago, they had an initial evaluation with one of the clinic's physical therapists; today is their first treatment session. They have previously received physical therapy at their local public clinic (CESFAM) and at this center for other issues.

**Scenario for Today's Session:** The patient is waiting for the student after receiving a 20-minute application of interferential current and moist hot packs. Despite this, they still report moderate pain with movement (4/10 on the Numeric Rating Scale).

**Script: Guidance for Questions**

**In response to the initial greeting:**

- Greet the student warmly and introduce yourself.
- Mention that your back still hurts despite the treatment you just received.
- Frequently touch or rub your lower back with the palm of your hand.

**In response to questions about your personal life (demographics):**

- Answer according to the provided data. You can improvise minor details (e.g., if your house has one or two floors).
- If asked about your support system, express that you feel lonely and unsupported.
- Explain that while your children are your motivation, you feel stressed because asking for their help with the house or business often leads to arguments.

**In response to questions about your daily function:**

- Explain that your limitations change from day to day.
- The moderate-to-severe low back pain forces you to move very slowly and use your arms for support.
- Emphasize a clear tendency to avoid movements like bending over, picking things up from the floor, or reaching overhead to stock shelves at your store.
- State that your back feels "fragile" and you don't feel capable of lifting anything heavier than five kilos.
- Express fear of movement (kinesiophobia). You are constantly preoccupied with your posture and try to avoid any movement that might worsen your back pain.

**When asked about your expectations for therapy:**

- State that your primary goal is for the pain to disappear completely.

**When asked about specific physical difficulties (e.g., strength, balance, mobility):**

- Emphasize that you *do* feel weaker, but you believe it is only because of the pain.
- Explain that the most difficult part is that nearly every movement hurts. This makes you constantly overthink how to move and worry about which movements might be harmful.

**When asked about your pain:**

- **Location:** Describe the pain as being in the lower back, with no radiating pain into the legs.
- **Intensity:** It varies from 4/10 to 8/10 on the Numeric Rating Scale (NRS) throughout the day.
- **Aggravating Factors:** The pain worsens when lifting heavy objects from the floor or reaching to place items on high shelves.
- **Characteristics / Timing:** The pain is constant, with a "squeezing" quality. It becomes a sharp, "stabbing" pain during movements with a large range of motion.

**Table S4.** *Post Hoc Pairwise Comparisons With Bonferroni Correction (k = 4 per outcome)*

| **Outcome** | **Comparison** | **Collaborative (mean ± SD)** | **Individual (mean ± SD)** | **Test statistic** | **P (unadj.)** | **P (Bonf.)** | **Cohen's d** |
| --- | --- | --- | --- | --- | --- | --- | --- |
|  | Collaborative: Pre vs Post | 25.14±4.15 vs 31.31±3.58 | — | *t*(11) = −5.231 | *P<.001* | *P=.001* | 1.510 |
| MTCCP | Individual: Pre vs Post | — | 26.61±3.96 vs 32.00±2.65 | *t*(10) = −5.979 | *P<.001* | *P<.001* | 1.803 |
|  | Collaborative vs Individual at Pre | 25.14 ± 4.15 | 26.61 ± 3.96 | *t*(21) = −0.827 | *P=.42* | *P=1.00* | 0.346 |
|  | Collaborative vs Individual at Post | 31.31 ± 3.58 | 32.00 ± 2.65 | *t*(21) = −0.502 | *P=.62* | *P=1.00* | 0.211 |
|  | Collaborative: Pre vs Post | 4.30±0.37 vs 4.34±0.37 | — | *t*(11) = −0.222 | *P=.83* | *P=1.00* | 0.064 |
|  | Individual: Pre vs Post | — | 4.53±0.34 vs 4.52±0.32 | *t*(10) = 0.082 | *P=.94* | *P=1.00* | 0.025 |
|  | Collaborative vs Individual at Pre | 4.30 ± 0.37 | 4.53 ± 0.34 | *t*(21) = −1.470 | *P=.16* | *P=.63* | 0.615 |
| CAT | Collaborative vs Individual at Post | 4.34 ± 0.37 | 4.52 ± 0.32 | *t*(21) = −1.198 | *P=.24* | *P=.98* | 0.502 |
|  | Collaborative: Pre vs Post | 117.69±6.00 vs 114.11±6.21 | — | *t*(11) = 1.637 | *P=.13* | *P=.52* | 0.472 |
| WAI-O | Individual: Pre vs Post | — | 120.09±7.92 vs 114.15±5.82 | *t*(10) = 2.558 | *P=.03* | *P=.11* | 0.771 |
|  | Collaborative vs Individual at Pre | 117.69 ± 6.00 | 120.09 ± 7.92 | *t*(21) = −0.786 | *P=.44* | *P=1.00* | 0.326 |
|  | Collaborative vs Individual at Post | 114.11 ± 6.21 | 114.15 ± 5.82 | *t*(21) = −0.015 | *P=.99* | *P=1.00* | 0.006 |

*Note.* Within-group comparisons (Pre vs Post) were conducted using paired-samples Student’s t-tests. Between-group comparisons (Collaborative vs Individual at each time point) were conducted using independent-samples Student’s t-tests. P (unadj.) = unadjusted two-tailed p-value; P (Bonf.) = Bonferroni-corrected p-value (α’ = .05/4 = .0125); — = not applicable (within-group comparison presents a single group). Cohen’s d computed as ΔM/SDΔ for within-group comparisons and ΔM/SDₚₒₒₗₛₜ for between-group comparisons. Magnitude benchmarks: small |d| ≈ .20, medium |d| ≈ .50, large |d| ≥ .80 (Cohen, 1988). MTCCP = Measurement Tool for Clinical Competencies in Physiotherapy; CAT = Communication Assessment Tool; WAI-O = Working Alliance Inventory—Observer Version.
